# *Limosilactobacillus fermentum* CCFM1126 attenuates osteoporosis by modulating the gut microbiota composition and fecal metabolites: A randomized, double-blind, placebo-controlled clinical trial

**DOI:** 10.1080/29933935.2026.2669712

**Published:** 2026-05-16

**Authors:** Yaru Liu, Jiani Pan, Leilei Yu, Chuan Zhang, Chengcheng Zhang, Qingwei Yao, Xingxing Chen, Xuesong Wang, Jianxin Zhao, Fengwei Tian, Qixiao Zhai, Wei Chen

**Affiliations:** a Wuxi Institute for Specialized Nutrition and Health, Co., Ltd., Wuxi, Jiangsu, China; b School of Food Science and Technology, State Key Laboratory of Food Science and Resources, Jiangnan University, Wuxi, Jiangsu, China; c School of Food Science and Engineering, Key Laboratory of Food Nutrition and Functional Food of Hainan Province, Hainan University, Haikou, China; d Department of Orthopedics, Affiliated Hospital of Jiangnan University, Wuxi, Jiangsu, China; e Wuxi School of Medicine, Jiangnan University, Wuxi, Jiangsu, China; f International Joint Research Laboratory for Probiotics at Jiangnan University, Wuxi, Jiangsu, China

**Keywords:** Probiotics, *Limosilactobacillus fermentum*, osteoporosis, gut microbiota, metabolites

## Abstract

Osteoporosis (OP) is a common systemic metabolic bone disorder characterized by reduced bone mass and deteriorated bone microarchitecture. *Limosilactobacillus fermentum* CCFM1126 has previously been shown to mitigate intestinal barrier dysfunction and modulate bone metabolism, thereby attenuating bone loss in OP rat models. To further evaluate its clinical efficacy, a double-blind, randomized, placebo-controlled trial was conducted. Additionally, the optimal intervention dose and potential bioactive components were investigated using ovariectomized (OVX) rat models and MC3T3-E1 pre-osteoblast cell assays. In the clinical trial, CCFM1126 supplementation significantly increased bone mineral density (BMD), elevated serum calcium and 25-hydroxyvitamin D levels, and reshaped gut microbiota composition by reducing the relative abundances of *Paraprevotella*, *Marvinbryantia*, *Ruminococcus 1*, and *Granulicatella*. In OVX rats, a dose of 10⁷ CFU/mL exerted minimal effects on bone morphology and metabolism, whereas doses ≥ 10⁸ CFU/mL led to significant improvements in bone structure and systemic metabolic indices, accompanied by favorable modulation of the gut microbial profile. The most pronounced effects were observed at 10⁹ CFU/mL. In MC3T3-E1 pre-osteoblasts, metabolites derived from CCFM1126 enhanced cell viability and upregulated the expression of key osteogenic genes. Among these, choline was identified as a potential bioactive compound contributing to the anti-osteoporotic effects of CCFM1126.

## Introduction

Osteoporosis (OP) is a widespread systemic metabolic bone disorder characterized by increased fracture risk, substantial trabecular bone loss, marked reductions in bone mass, deterioration of bone microarchitecture, and enhanced bone fragility.^1^ Current therapeutic strategies for OP include dietary modifications, lifestyle interventions, and pharmacological treatments.^2^^,^^3^ In the early stages of the disease, consuming nutrient-rich foods such as those high in calcium and vitamin D can enhance calcium absorption and improve bone mineral density (BMD). Moderate physical activity and outdoor exercise facilitate vitamin D synthesis, bolster muscle strength and balance, and stimulate bone tissue metabolism.^4-7^ Recently, numerous novel alkaline phosphatase inhibitors have been designed and synthesized, primarily through structural modifications of existing drugs or natural products to develop derivatives and analogues with anti-osteoporotic properties.^8^^,^^9^ These compounds have demonstrated promising alkaline phosphatase (ALP) activity *in vitro* and *in vivo*, along with the ability to inhibit interferon-induced tartrate-resistant acid phosphatase (TRACP) activity, thereby promoting osteoblast differentiation and mineralization while preventing excessive bone resorption.^9^^,^^10^ However, long-term or high-dose administration of these agents may lead to adverse effects. For instance, calcium supplementation may result in hypercalcemia, bisphosphonates can cause gastrointestinal discomfort, and estrogen replacement therapy may increase the risk of breast cancer.^11-13^ With the in-depth study of the “bone-gut” axis, increasing attention has been directed toward the roles of dietary supplements, probiotics, and prebiotics in regulating bone metabolism.^14-16^ Functional foods and probiotic or synbiotic formulations based on dairy and plant matrices, as well as brewer's industry by-products, have been explored as strategies to modulate the gut microbiota and systemic metabolism, potentially impacting bone health. For example, brewer’s spent grain has been shown to enhance probiotic survival under simulated gastrointestinal conditions, while plant-derived by-products can promote microbiota modulation and short-chain fatty acid production.^17^^,^^18^

Probiotics are live microorganisms that, when administered in adequate amounts, confer health benefits to the host. In animal models, specific *Lactobacillus* species, including *L. acidophilus*, *L. helveticus*, and *L. curvatus*, have been shown to reduce inflammation in the gut and bones, decrease gut permeability, prevent bone loss, and alleviate OP.^19-21^ Building on these mechanistic insights, *Lactobacillus* and related lactic acid bacteria have also been applied in dairy and plant-based fermented matrices as probiotic vehicles, demonstrating functional viability and potential translational relevance,^17^^,^^18^^,^^22^^,^^23^ with growing evidence that such matrix-based delivery systems can shape gut microbiota composition and metabolite profiles, thereby influencing host metabolic responses. Clinical evidence also supports the role of probiotics in modulating bone metabolism through gut microbiota regulation, offering significant potential as an adjuvant treatment for OP—particularly in patients who are allergic to standard medications or have comorbidities that preclude the use of conventional anti-osteoporotic drugs.^24^^,^^25^ In a previous meta- analysis, three randomized double-blind trials were conducted, involving 365 patients with osteopenia who were allocated into placebo and probiotic groups; the lumbar BMD levels in the probiotic supplementation group were significantly higher compared to those in the placebo group.^26^

Nonetheless, prior research findings still exhibit certain limitations. Most randomized controlled trials of probiotics for OP published to date have primarily focused on evaluating the efficacy of probiotics, while their dose-response relationships and potential mechanisms of action remain largely understudied. Moreover, while several animal studies have reported bone-protective effects of probiotics, few studies have systematically integrated well-designed clinical intervention data with mechanistic validation at the microbial and metabolic levels. In particular, studies focusing on a single, well-characterized probiotic strain and combining clinical efficacy with multi-level mechanistic evidence are still scarce, which limits causal interpretation and translational relevance. *Limosilactobacillus fermentum* CCFM1126 has been certified to mitigate gut barrier damage induced by TNF-α exposure, regulates bone structural indices such as BMD and bone volume/total volume (BV/TV) in OP rats, improves bone metabolic markers like Bone alkaline phosphatase, normalizes serum inflammatory factor TNF-α levels, and effectively inhibits the downregulation of gut tight junction protein mRNA expression in OP rats, thereby ameliorating bone loss.^27^ However, its clinical efficacy in humans and the mechanisms underlying its potential bone-protective effects remain to be fully elucidated. Therefore, the present study was designed to address these gaps through a hierarchical and integrative approach. A randomized, double-blind, placebo-controlled clinical trial was first conducted to evaluate the efficacy of a single-dose intervention with *L. fermentum* CCFM1126 in individuals with osteoporosis. Subsequently, 16S rRNA gene sequencing and untargeted metabolomics were employed to characterize gut microbiota alterations and metabolic features associated with the observed clinical outcomes. In parallel, complementary animal and *in vitro* experiments were performed at multiple dosage levels to explore potential dose-dependent effects and underlying biological mechanisms, thereby providing mechanistic support for the clinical observations. Collectively, this integrative “clinical validation–mechanistic analysis–experimental support” strategy advances prior probiotic research in osteoporosis by bridging clinical efficacy with biological plausibility while maintaining clear boundaries between clinical and experimental findings.

## Methods

### Culture of *L. fermentum*

Strains of *L. fermentum* CCFM1126 (*L. fermentum* 51L) and *L. fermentum* FSDHZD13L1 were isolated from adult fecal samples. Previous studies have demonstrated that CCFM1126 exhibits the potential to alleviate osteoporosis in rats, whereas FSDHZD13L1 does not possess this effect and is therefore utilized as a negative control strain.^27^ In the present clinical trial, only *L. fermentum* CCFM1126 was used for probiotic supplementation. The placebo product contained maltodextrin only and did not include any viable microorganisms, while the probiotic product comprised maltodextrin supplemented with *L. fermentum* CCFM1126 at a viable cell count of 5 × 10^9^ CFU per pack.^14^^,^^17^^,^^28^^,^^29^ Both products were manufactured to food-grade standards and presented as powders with identical appearance and packaging. The recommended dosage was 2 g per pack, administered twice daily.

### Ethical approvement

This study received ethical review and approval from the Medical Ethics Committee of Jiangnan University in Wuxi, Jiangsu Province (approval number: JNU20230901IRB03) and was registered with the Chinese Clinical Trial Registry (registration number: ChiCTR2300076115), adhering to the ethical guidelines outlined in the Declaration of Helsinki. This study employed a double-blind, parallel-group randomized controlled trial design. All participants provided informed consent prior to the commencement of the study, and all study records will be securely maintained at the School of Food Science and the Affiliated Hospital of Jiangnan University. Any public dissemination of the study results will ensure that no personal information of the participants is disclosed.

### Clinical trial design

This clinical trial was conducted from October 2023 to June 2024. Participants included individuals aged 45 y and older, irrespective of gender, who met at least one of the following criteria: A prior diagnosis of osteoporosis or osteopenia confirmed by bone mineral density (BMD) testing; Identification by healthcare professionals as being at risk for OP. Exclusion criteria were as follows: A history of serious diseases (e.g., malignant tumors) or gastrointestinal disorders; presence of secondary or idiopathic OP; use of any hormones, OP medications, or other drugs that may interfere with bone health during the study period; consumption of antibiotics or probiotic products within one month prior to study enrollment; participation in other clinical trials.

The entire clinical trial lasted for a duration of three months. Upon agreeing to participate in the study, subjects were required to sign an informed consent form and complete both their personal information forms and the IOF osteoporosis risk assessment questionnaire. At both baseline and the 3-month follow-up, participants underwent a standardized physical examination, during which the following parameters were measured: body weight and height (for calculation of body mass index, BMI), blood pressure, heart rate, and BMD. Fecal and whole blood samples were also collected for subsequent microbiota and metabolomic analyses. After taking probiotics or placebo for 3 months, a second physical examination was scheduled. Participants had the right to withdraw from or terminate their involvement in the study at any time. If participants experienced any health issues or significant physiological changes during the study, they were advised to discontinue their participation. Participants who were unable to attend scheduled physical examinations or provide feces or blood samples were excluded from further participation. Throughout the trial period, participants were required to refrain from using other probiotic products or antibiotics and to maintain consistent dietary habits.

### Serum measures

Whole blood samples were collected at two time points: at baseline (before intervention) and after the 3-month intervention period. All samples were obtained in the morning following an overnight fast. Whole blood samples were collected by the staff of the Physical Examination Center at the Affiliated Hospital of Jiangnan University into coagulation-promoting tubes. Subsequently, the samples were centrifuged at 4 °C and 3000 r/min for 15 min. Blood collection was performed on the same day as the physical examination and BMD assessment. Serum calcium, phosphorus, magnesium, chlorine, sodium, potassium, and 1,25(OH)_2_VD were determined by the physical examination Center of the Affiliated Hospital of Jiangnan University. Serum TRACP-5b, OPG, TNF-α, and IL-1β were detected by an enzyme-linked immunosorbent assay kit.

### Gut microbiota analysis

Fecal microbiota DNA was extracted using the Fast DNA Spin Kit for feces (MP Biomedicals, Santa Ana, CA, USA) following the manufacturer's protocol. Specific regions of 16S rRNA gene V3 to V4 were amplified by PCR using priors 341F: 5ʹ-CCTAYGGGRBGCASCAG-3ʹ, 806R: 5ʹ-GGACTACNNGGGTATCTAAT-3ʹ. The quantitative PCR products were isolated via agarose gel electrophoresis. Subsequently, DNA was extracted and purified following the manufacturer's protocol for the DNA glue recovery kit, and its concentration was quantified. The purified amplicons were then sequenced on the Illumina MiSeq PE300 platform. Drawing upon prior reference protocol, QIIME2 2022.2 was utilized to process the 16S rRNA gene sequencing data, thereby obtaining raw sequence information and subsequently optimizing and refining the data.^26^ And open reference Amplicon sequence variants (ASVs) were used to assign the reads. Sequence alignment was performed using the Silva Bacterial Database. We used the R vegan, ape, ggpubr, ggplot2 libraries, and GraphPad Prism 8 for analysis and visualization, and Python's NumPy and SciPy libraries for ASV-tracking. Microbial co-abundance analysis is done using the online website chiplot (https://www.chiplot.online/#Bar-plot). Spearman correlation and heatmap analysis and visualization was performed using the OmicStudio tools (at https://www.omicstudio.cn/tool).

### Metabolomics detection

20 mg feces sample from the subject was weighed into a 1.5 mL centrifuge tube and subsequently homogenized with 200 μL of deionized water using a high-throughput tissue grinder. To obtain strain-derived metabolites for comparative metabolomic analysis, activated *L. fermentum* (after two generations) was inoculated into MRS medium at an inoculation rate of 2%. Following a 12-h incubation period, 1 mL of the bacterial culture was harvested and subjected to centrifugation at 4 °C at 8000 r/min for 5 min to obtain the supernatant. In accordance with a previously reported method,^30^ 200 μL samples (feces supernatant, serum, and bacterial fermentation supernatant, respectively) were transferred to 1.5 mL centrifuge tubes for metabolite extraction. A mixture of methanol and acetonitrile was introduced into the homogenate, followed by ice-bath sonication to promote protein precipitation. Subsequently, the sample was centrifuged, and the supernatant obtained was dried using vacuum centrifugation. The dried residue was then redissolved in an acetonitrile-water solution. Metabolites were analyzed by Q Exactive liquid chromatography-mass in full scan mode. The resulting data were processed using Compound Discoverer 3.3.

### Animal experimental design

The SPF female Sprague-Dawley rats (12 weeks old, with an average weight of approximately 250 g) utilized in this study were procured from Beijing Vital River Laboratory Animal Technology Co., Ltd. The animals were maintained in a barrier-controlled environment set at a temperature of 20 °C–24 °C and relative humidity of 40%–60%, under a 12-h light-dark cycle. This experimental protocol was approved by the Ethics Committee of Jiangnan University (Ethics No. JN. No 20230515S0700930[184]), and all procedures strictly adhered to the guidelines for animal experimentation and welfare. During the entire experimental period, all rats were fed a standard SPF-grade maintenance diet supplied by the Experimental Animal Center of Jiangnan University. The diet was formulated according to the national standards for laboratory animal feed (GB 14924.3-2010) and contained approximately 1.0% calcium (w/w) and 0.6%–0.8% phosphorus (w/w), which are sufficient to meet the nutritional requirements for normal bone metabolism in adult rats. A phytoestrogen-free or modified calcium diet was not used. All experimental groups received the same diet to minimize potential dietary confounding effects.

A total of 64 rats were randomly assigned to eight groups: the control (CON, sham-operated control) group, the model (MOD, ovariectomized osteoporosis model) group, positive control (POS) group, negative control (NEG) group, 10^7^ CFU/mL group, 10^8^ CFU/mL CCFM1126 group, 10^9^ CFU/mL group, and 10^10^ CFU/mL CCFM1126 group. After a 7-d acclimatization period, bilateral ovariectomy was performed in all groups except the control (CON) group under 2% isoflurane anesthesia. The CON group served as the sham-operated control, in which rats underwent the same surgical procedures as the ovariectomized groups, but without bilateral ovariectomy. Post-surgery, all rats received subcutaneous injections of penicillin at a dose of 2 × 10^5^ U/kg body weight for three consecutive days to prevent infection. Two weeks after recovery, gavage administration commenced with a volume of 1.5 mL per rat, continuing for 90 d. The CON and MOD groups received sterile saline, the POS group received a 0.1 mg/kg body weight estradiol (E_2_) solution, and the NEG group received a 10^9^ CFU/mL *L. fermentum* FSDHZD13L1 suspension. The 10^7^ CFU/mL, 10^8^ CFU/mL, 10^9^ CFU/mL, and 10^10^ CFU/mL groups received corresponding doses of *L. fermentum* CCFM1126 suspension. Daily observations were conducted to monitor the activity and condition of the rats, which had free access to food and water throughout the experiment. At the end of the experimental period, rats were anesthetized with 3%–4% isoflurane until a surgical level of anesthesia was achieved, as confirmed by the complete loss of pedal withdrawal reflex. Blood samples were then collected via cardiac puncture under deep anesthesia. Following blood collection, rats were euthanized by isoflurane overdose (5% inhalation). Death was confirmed by cessation of respiration and heartbeat. Intestinal contents and bone tissues were subsequently collected.

### Determination of bone structure in rats

Bone microarchitecture was analyzed using micro-computed tomography (micro-CT). The distal end of the left femur from each rat was positioned within the micro-CT imaging system for X-ray scanning. The samples were scanned under the following conditions: X-ray voltage of 70 kV, current of 114 μA, and an exposure time of 300 ms per projection. During scanning, the samples were rotated through a full 360-degree cycle, and the voxel size (field of view) was set at 36 μm. The acquired images were subsequently imported into Analyze 12.0 software to reconstruct three-dimensional models of the rat femurs and to quantify BMD, BV/TV, trabecular separation (Tb.Sp), and trabecular thickness (Tb.Th) at the distal femoral region.

### Pathological section of rat femur

The left femur tissues of the rats were meticulously cleansed with normal saline and immediately transferred into a paraformaldehyde fixation solution for a 24-h period. Subsequently, the decalcified bone tissues were subjected to decalcification using an EDTA chelating agent. The decalcified specimens were sectioned to a thickness of 3 mm and placed into embedding cassettes. The tissue sections were stained with Hematoxylin and Eosin. Finally, the prepared sections were scanned using a Pannoramic MIDI digital slide scanner to assess the pathological changes in the femur tissue.

### Cell proliferation assay

100 μL of MC3T3-E1 cells, adjusted to a density of 5 × 10^4^ cells/mL, were seeded into 96-well plates and incubated for 24 h. Following the incubation period, the cell culture supernatant was aspirated. The pH of the *L. fermentum* supernatant was adjusted to 7.0 and subsequently filtered through a 0.22 µm filter membrane before being added to α-MEM complete medium. MC3T3-E1 cells were then exposed to varying concentrations of the *L. fermentum* supernatant for 24, 48, and 72 h. After each incubation period, the culture medium was removed, and 100 μL of fresh medium along with 10 μL of CCK-8 solution were added. The plate was incubated for 2 h, after which the absorbance at 450 nm was measured for each well.

### Determination of gene expression related to bone metabolism

The cells were seeded in 6-well plates at a density of 1.05 × 10^5^ cells/mL (2 mL per well) and cultured at 37 °C for 48 h until approximately 80% confluence was reached. After absorbing the supernatant of cell culture medium, the pH of bacterial supernatant was adjusted to 7.0, followed by filtration through a 0.22 µm filter membrane before being added to α-MEM complete medium. A control group was established using α-MEM without the bacterial supernatant. Cells were then incubated with the α-MEM complete culture medium containing the bacterial supernatant at 37 °C for 24 h, after which they were washed twice with PBS buffer. RNA extraction and reverse transcription into cDNA were performed as described by Xu et al. ^31^. Subsequently, RT-qPCR was performed on a BioRad-CFX384 machine (Bio-Rad, California, USA) using SYBR Green Supermix. The PCR reaction was performed in a total volume of 10 μL. The thermal cycling consisted of 95 °C for 30 s, and 40 cycles of 95 °C for 5 s, 60 °C for 30 s. After the PCR a melting curve (65 °C ~95 °C, increment 0.5 °C) was generated to check the specificity of the amplified fragment. The primer sequences used are listed in Table 1.

**Table 1.** Primers sequences used for real-time quantitative polymerase chain reaction.

Primer name	Upstream primer	Downstream primer
GAPDH	5ʹ-GCAAAGTGGAGATTGTTGCCAT-3ʹ	5ʹ-CCTTGACTGTGCCGTTGAATTT-3ʹ
ACP5	5ʹ-GCCAAGATGGATTCATGGGTGG-3ʹ	5ʹ-CAGAGACATGATGAAGTCAGCG-3ʹ
OPG	5ʹ-CCTTGCCCTGACCACTCTTAT-3ʹ	5ʹ-CACACACTCGGTTGTGGGT-3ʹ
RANKL	5ʹ-AGCCGAGACTACGGCAAGTA-3ʹ	5ʹ-AAAGTACAGGAACAGAGCGATG-3ʹ
β-catenin	5ʹ-TGGTGACAGGGAAGACATCA-3ʹ	5ʹ-TGGTGACAGGGAAGACATCA-3ʹ
RUNX-2	5ʹ-CCGAACTGGTCCGCACCGAC-3ʹ	5ʹ-CTTGAAGGCCACGGGCAGGG-3ʹ
ALP	5ʹ-CCAACTCTTTTGTGCCAGAGA-3ʹ	5ʹ-GGCTACATTGGTGTTGAGCTTTT-3ʹ

### Data statistics and analysis

Microsoft Excel (version 2021), GraphPad Prism (version 9.5), and R software (version 3.4) were used to organize and statistically analyze the experimental data, which were expressed as mean ± standard deviation (SD). The non-parametric Kruskal–Wallis test was used for single-factor analysis. Wilcoxon paired test was used for paired comparison between two groups of the same subject, and Wilcoxon unpaired test was used for unpaired comparison between two groups of different subjects. Using Bray–Curtis dissimilarity calculation, Principal co-ordinates analysis (PCoA) was performed on the distance matrix. Linear discriminant analysis Effect Size (LEfSe) was used to assess differences in the relative abundance of microbial features. OPLS-DA multivariate analysis was performed for both groups of metabolites using SIMCA 14.1 and Variable importance in projection (VIP) was calculated. *p*-values and FC values were obtained using the *t*-test to calculate significant differences between the two groups of metabolites. Using MetaboAnalyst (https://www.metaboanalyst.ca/) pathway enrichment analysis was carried out on the difference of metabolites. QIIME2 2022-2 was used for correlation analysis, and “corrplot” and “ggplot2” of R software (https://www.r-project.org/) were used to calculate and plot the Spearman correlation coefficients. *, **, and *** indicate statistical significance, corresponding to *p* < 0.05, <0.01, and <0.001, respectively.

## Result

### Demographic information

A total of 44 participants who met the inclusion criteria were enrolled in this clinical study. Thirteen participants withdrew from the trial for various personal reasons, including non-compliance with probiotic intake schedules, concurrent antibiotic use, and loss to follow-up. We have systematically analyzed the baseline characteristics of the remaining 31 participants and randomly allocated them into placebo and probiotic groups (detailed analysis procedure was shown in Tables 2 and S1). These data provide a comprehensive overview of the participants' physical conditions and changes in relevant indicators. No significant differences were observed between the two groups.

**Table 2.** Population queue basic information.

Basic information	Placebo group (*N* = 14)	CCFM1126 group (*N* = 17)	*p-*value
Age, mean ± SD	64.5 ± 9.89	64.76 ± 7.97	0.6732
Gender (*n*, %) female	13 (92.86%)	16 (94.12%)	>0.9999
male	1 (7.14%)	1 (5.88%)	
BMI, mean ± SD	23.51 ± 3.19	23.97 ± 3.14	0.8220
Risk score, mean ± SD	3.786 ± 1.76	3.529 ± 2.00	0.6796

Note: the risk score (0–18) was calculated using the International Osteoporosis Foundation Osteoporosis Risk Assessment, with Body Mass Index (BMI) calculated as weight (kg)/height^2^ (m).

### Effects of *L. fermentum* CCFM1126 on physiological and biochemical indexes in OP patients

The effects of *L. fermentum* CCFM1126 on OP patients were evaluated using serum physiological and biochemical indices, as detailed in Table 3. At baseline, no significant differences were observed in any biochemical markers between the placebo and CCFM1126 groups (Wilcoxon unpaired test, *p* > 0.05). After a 3-month intervention period, the serum calcium level in the CCFM1126 group (2.471 ± 0.06 mmol/L) was significantly higher than that in the placebo group (2.421 ± 0.08 mmol/L, Wilcoxon unpaired test, *p* = 0.0226). Although other serum biochemical markers showed improvement after 3 months of intervention with *L. fermentum* CCFM1126 compared to the placebo, these changes were not statistically significant (Wilcoxon unpaired test, *p* > 0.05). Our findings indicate that continuous administration of CCFM1126 for 3 months resulted in a significant increase in serum vitamin D levels (Wilcoxon paired test, *p* = 0.0040) and OPG content (Wilcoxon paired test, *p* = 0.0206) in OP patients relative to baseline. Conversely, TRACP levels were significantly reduced (Wilcoxon paired test, *p* = 0.0079).

**Table 3.** Effects of *L. Fermentum* CCFM1126 on physiological and biochemical indexes in OP patients.

Indexes	Placebo group (*N* = 14)		CCFM1126 group (*N* = 17)		*p-*value		
	Pre	Post	Pre	Post	Placebo-pre & Placebo-post^#^	CCFM1126-pre & CCFM1126-post^#^	Placebo-post & CCFM1126-post^##^
Magnesium (mmol/L)	0.8471 ± 0.08	0.8343 ± 0.07	0.8788 ± 0.03	0.8782 ± 0.05	0.3311	0.7249	0.2020
Chlorine (mmol/L)	105.2 ± 2.72	104.6 ± 2.85	104.8 ± 2.25	104.7 ± 1.94	0.2744	0.9172	0.9766
Calcium (mmol/L)	2.429 ± 0.06	2.421 ± 0.08	2.450 ± 0.08	2.471 ± 0.06	0.4259	0.4294	0.0226*
Sodium (mmol/L)	142.0 ± 2.24	141.7 ± 2.45	141.1 ± 1.94	140.8 ± 1.61	0.4722	0.4952	0.1637
Potassium (mmol/L)	4.052 ± 0.35	3.889 ± 0.39	4.038 ± 0.38	4.025 ± 0.30	0.5525	0.8265	0.2749
Phosphorus (mmol/L)	1.219 ± 0.19	1.178 ± 0.12	1.191 ± 0.18	1.165 ± 0.11	0.2163	0.7370	0.6173
Vit D (ng/mL)	20.38 ± 6.87	20.47 ± 7.04	18.88 ± 5.18	22.47 ± 6.97	0.9515	0.0040**	0.5437
ALP (U/L)	84.21 ± 17.04	82.71 ± 17.32	88.18 ± 17.04	82.94 ± 17.53	0.4636	0.1292	0.9766
TRACP (U/L)	1.754 ± 0.45	1.559 ± 0.35	1.770 ± 0.36	1.418 ± 0.30	0.0906	0.0079**	0.3307
OPG (ng/L)	1.561 ± 0.95	1.662 ± 0.77	1.595 ± 0.61	1.749 ± 0.66	0.3998	0.0206*	0.3508
TNF-α (ng/L)	233.1 ± 79.37	243.8 ± 53.45	231.3 ± 42.47	225.8 ± 59.86	0.4856	0.8086	0.3933
IL-1β (ng/L)	4.192 ± 1.93	4.037 ± 1.73	3.925 ± 1.57	3.280 ± 1.49	0.5824	0.2683	0.1964

Note: results are expressed as mean ± SD; ^#^means Wilcoxon paired test, ## means Wilcoxon unpaired test;

^*^ and ** indicate statistical significance, corresponding to *p* < 0.05 and <0.01, respectively.

### Effect of *L. fermentum* CCFM1126 on BMD in OP patients

**Figure 1.**
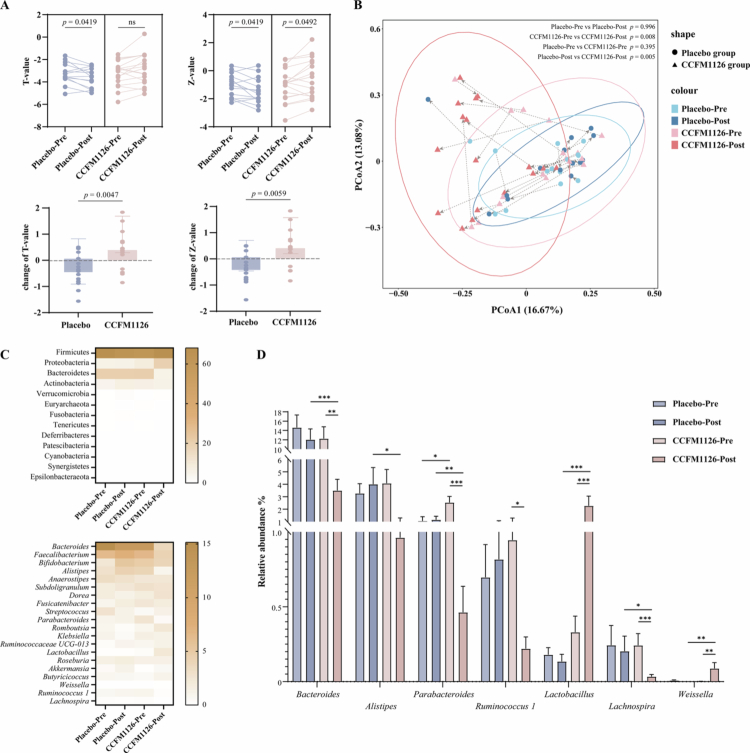
Effects of *L. fermentum* CCFM1126 on BMD and gut microbiota diversity in OP patients. (A) The T-value and Z-value, along with their variations. (B) PCoA analysis of microbiota based on Bray–Curtis difference. Each scatter plot represents an individual sample, while the line connects the baseline period to the end period for the same subject. Group differences were assessed using ANOSIM (Analysis of Similarities) based on Bray–Curtis distances. (C) Comparison of relative abundance of microbiota at phylum level and genus level. (D) Comparison of relative abundance of core differential strains. Using Wilcoxon unmatched test, *, **, and *** indicate statistical significance, corresponding to *p* < 0.05, < 0.01, and < 0.001, respectively.

The BMD of OP patients was measured using dual-energy X-ray absorptiometry (DXA) with a GE Lunar Prodigy Advance system (GE Healthcare, USA). The OP patients demonstrated a significant enhancement in BMD after a three-month intervention with *L. fermentum* CCFM1126 compared to the placebo group (Figure 1A). Specifically, following placebo administration, both T and Z scores exhibited a significant decline relative to baseline levels (Wilcoxon paired test, *p* = 0.0419), indicating no improvement in OP status. Conversely, after administration of CCFM1126, there was a significant increase in the Z score (Wilcoxon paired test, *p* = 0.0492), while the T score showed an upward trend, albeit not statistically significant (Wilcoxon paired test, *p* > 0.05). Compared to the placebo group, the change in T score in the probiotic group (0.39 ± 0.69) was significantly greater than that in the placebo group (−0.39 ± 0.61, Wilcoxon unpaired test, *p* = 0.0047). Similarly, the change in Z score in the CCFM1126 group (0.41 ± 0.68) was significantly higher than that in the placebo group (−0.36 ± 0.58, Wilcoxon unpaired test, *p* = 0.0059).

### Effect of *L. fermentum* CCFM1126 on gut microbiota in OP patients

As shown in Figure S1, intervention with *L. fermentum* CCFM1126 significantly reduced the Shannon index (Wilcoxon paired test with baseline of probiotics, *p* = 0.0488; Wilcoxon unpaired test with the endpoint of placebo, *p* = 0.0166) and Simpson index (Wilcoxon paired test with baseline of probiotics, *p* = 0.0093; Wilcoxon unpaired test with the endpoint of placebo, *p* = 0.0069) of the gut microbiota. Compared to the baseline period, no significant alteration was observed in the Shannon index or Simpson index of gut microbiota within the placebo group (Wilcoxon paired test, *p* > 0.05). PCoA based on Bray–Curtis dissimilarities was employed to visualize the β diversity of gut microbiota in both groups, both prior to and following the intervention (Figure 1B). The PERMANOVA analysis revealed that the gut microbiota structure of subjects supplemented with *L. fermentum* CCFM1126 for a period of three months exhibited significant alterations (*p* < 0.05). The gut core microbial composition of the two subject groups was analyzed both before and after the intervention, as illustrated in Figure 1C and D. Following CCFM1126 intervention, there was a significant increase in the relative abundance of *Proteobacteria* (Wilcoxon unpaired test, *p* < 0.001) compared to the placebo group, while the relative abundance of *Bacteroides* showed a significant decrease (Wilcoxon unpaired test, *p* < 0.001). Consistent results were observed when comparing the phylum level changes before and after CCFM1126 intervention (Wilcoxon unpaired test, *p* < 0.01). At the genus level, the gut microbiota of probiotic-treated subjects exhibited a significantly higher relative abundance of *Lactobacillus* and *Weissella*compared to the placebo group. Conversely, there was a notable decrease in the relative abundances of *Bacteroides*, *Paraprevotella*, *Alistipes*, *Marvinbryantia*, *Ruminococcus 1*, and *Lachnospira* (Wilcoxon unpaired test, *p* < 0.05).

### Effects of *L. fermentum* CCFM1126 on metabolites in OP patients

**Figure 2.**
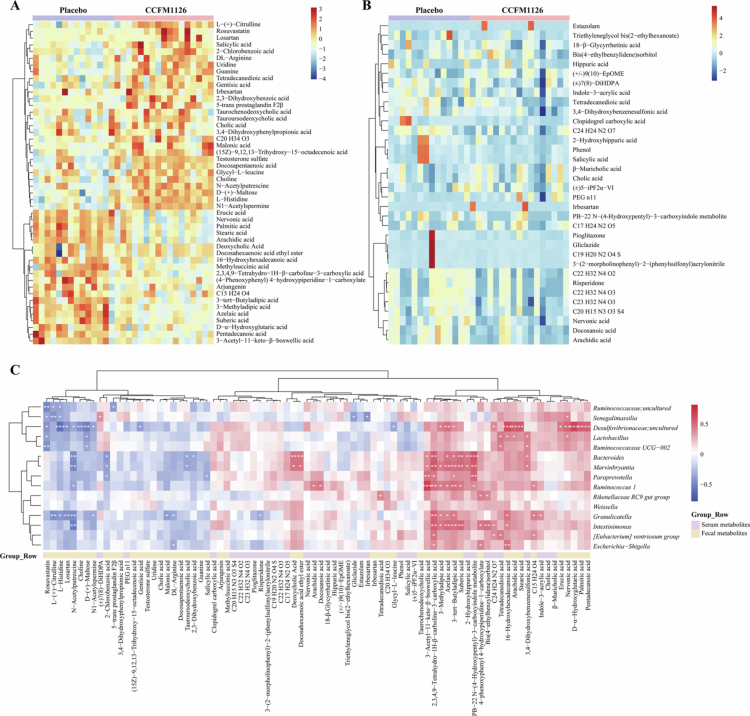
Relative levels of differential metabolites between the two groups at the intervention end point. (A) Heatmap showing the relative levels of significantly altered fecal metabolites between the placebo and CCFM1126 groups at the end of the intervention. Metabolites were screened based on multivariate and univariate criteria (VIP > 1, FC > 1.5 or <0.67, *p* < 0.05), and data are presented as row-wise standardized values (z-scores). (B) Heatmap of significantly altered serum metabolites between the two groups at the intervention endpoint using the same selection criteria as in (A). (C) Correlation analysis between differential gut bacterial genera and differential metabolites at the end of the intervention. Hierarchical clustering was performed using Ward's D2 method, and correlations were calculated using Spearman’s rank correlation analysis. Red and blue colors indicate positive and negative correlations, respectively. Only significant correlations are shown. *, **, and *** indicate statistical significance at *p* < 0.05, *p* < 0.01, and *p* < 0.001, respectively. For metabolites annotated with molecular formulas only, detailed compound names are provided as follows: C23H32N4O3 stands for 1-(4-methyl-1-piperazinyl)-2-[(3 R,4S)-3-{[5-(phenoxymethyl)-1,2-oxazol-3-yl] methyl}-4-piperidinyl] ethanone, C19H20N2O4 S stands for N1-(4-isopropylphenyl)-4-(2, 5-dioxotetrahydro-1h-pyrrol-1-yl) benzene-1-sulfonamide, C22H32N4O3 stands for (2S,5aS,8aR) -6-benzyl-1-methyl-2-[3-(4-morpholinyl)-3-oxopropyl] octahydropyrrolo [3, 2-e][1,4] diazepin-5(2H)-one, C20H15N3O3S4 stands for 4-{5-[(4-methylphenyl) methylene]-4-oxo-2-thioxo-1,3-thiazolan 3-yl}-N-(1,3-thiazol-2-yl) benzenesulfonamide, C22H32N4O2 stands for N-({(1S,4S,6S) -6-isopropyl-3-methyl-4-[2-oxo-2-(1-pyrrolidinyl) ethyl]-2-cyclohexen-1-yl}methyl)-2-pyrazinecarboxamide, C24H24N2O7 stands for 3-{[4-(1, 3-benzodioxol-5-ylmethyl) piperazino] carbonyl}-6, 7-dimethoxy-2h-chromen-2-one, C17H24N2O5 stands for N-{4-[(2R,3R)-3-(hydroxymethyl) -4-isopropyl-5-oxo-2-morpholinyl] phenyl}-2-methoxyacetamide, C15H24O4 stands for 3-[4-methyl-1-(2−methylpropanoyl)-oxocyclohexyl] butanoic acid, C20H34O3 stands for 5-[(1S,2R,4aR)-5-(hydroxymethyl)-1,2,4-trimethyl-1,2,3,4, 4A,7,8,8a-octahydro-1-naphthalenyl]-3-methylpentanoic acid.

Following a 3-month intervention with *L. fermentum* CCFM1126, significant differences were observed in the fecal metabolites of subjects compared to the placebo group (Figure 2A and Table S2). After conditional screening (VIP > 1, FC > 1.5 or FC < 0.67, *p* < 0.05), it was observed that CCFM1126 significantly increased the fecal content of several metabolites in subjects, including N1-acetylarginine, malonic acid, 2-chlorobenzoic acid, N-acetyl-1,4-diaminobutane, L-histidine, D-maltose, choline, arginine, salicylic acid, L-citrulline, gentisic acid, tetradecanedioic acid, docosapentaenoic acid, 2,3-dihydroxybenzoic acid, N-acetylbutyric acid, and promoted the absorption of various substances such as uridine, glycinyl-L-leucine, guanine, tauroursodeoxycholic acid, taurocholic acid, palmitic acid, 3-methyladipic acid, erucic acid, 3,4-dihydroxyphenylpropionic acid, neric acid, 16-hydroxypalmitic acid, methylsuccinic acid, azelaic acid, 3-acetylglycyrrhetinic acid, octamethylene acid, deoxycholic acid, cholic acid, arachidic acid, D-2-hydroxyglutaric acid, and ethyl docosahexaenoate. An analysis utilizing the Kyoto Encyclopedia of Genes and Genomes (KEGG) pathway revealed that, in comparison to a placebo, CCFM1126 may influence lipid synthesis and metabolism, amino acid synthesis and metabolism, cellular energy metabolism, and glucose metabolism. Furthermore, we conducted a comparative analysis of feces metabolomics before and after probiotic intervention, identifying significant differences. Under identical screening conditions, 11 significantly up-regulated and 11 significantly down-regulated differential metabolites were observed. Additionally, CCFM1126 demonstrated potential regulatory effects on the biosynthesis pathways of phenylalanine, tyrosine, and tryptophan. Whether before or after probiotic intervention, or the metabolite differences between the probiotic group and the placebo group at the end of the intervention, these changes are evident in feces samples (Tables S2 and S3). Specifically, N1-acetylspermine, malonic acid, choline, uridine, guanine, and L-citrulline were significantly increased following the administration of CCFM1126. Conversely, tauroursodeoxycholic acid, palmitic acid, erucic acid, nervonic acid, 16-hydroxyhexadecanoic acid, and arachidic acid exhibited significant decreases (*p* < 0.05).

*L. fermentum* CCFM1126 significantly altered serum metabolites in OP patients, with results presented in Figure 2B, Tables S4 and S5. After a 3-month intervention period, significant differences in serum metabolites were observed between the placebo and CCFM1126 groups. Specifically, we identified 10 metabolites with increased levels and 24 metabolites with decreased levels by screening for VIP values (VIP > 1) and fold change (FC) values (FC > 1.5 or FC < 0.67). KEGG pathway analysis revealed that CCFM1126 intervention influenced linoleic acid metabolism, unsaturated fatty acid biosynthesis, and primary bile acid biosynthesis compared to the placebo group. Additionally, we examined changes in the serum metabolome within the probiotic group pre- and post-intervention. Under the same screening criteria, we identified 8 metabolites with increased levels and 14 metabolites with decreased levels. Besides the three pathways, CCFM1126 also impacted caffeine metabolism, citric acid cycle, alanine, aspartate and glutamate metabolism, glyoxylate and dicarboxylate metabolism, and sphingolipid metabolism.

### Association analysis between gut microbiota and differential metabolites

To further elucidate the interaction between gut microbiota and host metabolism, we investigated the association between gut microbiota composition and both fecal and serum metabolites at the intervention endpoint in both groups (Figure 2C). The differential fecal metabolites exhibit significant correlations with host metabolic changes. Specifically, *Lactobacillus*, *Bacteroides*, *Marvinbryantia*, *Ruminococcus 1*, *Granulicatella*, and *Intestinimonas* display notable associations with various metabolites. The relative abundance of *Lactobacillus* was significantly positively correlated with the levels of 16-hydroxypalmitic acid, nervonic acid, and arachidonic acid (0.37 < R < 0.39, *p* < 0.05). *Bacteroides* and *Marvinbryantia* showed significant positive correlations with ethyl docosahexaenoate, deoxycholic acid, 3-acetylglycyrrhetinic acid, azelaic acid, octadecanoic acid, and 3-tert-butyladipic acid (0.37 < R < 0.49, *p* < 0.05), while exhibiting significant negative correlations with tauroursodeoxycholic acid, 2-chlorobenzoic acid, and N-acetyl-1,4-diaminobutane (−0.52 < R < −0.37, *p* < 0.05). *Ruminococcus 1* demonstrated significant positive correlations with 3-acetylglycyrrhetinic acid, 3-tert-butyladipic acid, 3-methyladipic acid, azelaic acid, and octadecanoic acid (0.43 < R < 0.65, *p* < 0.05), as well as a significant negative correlation with N-acetyl-1,4-diaminobutane (R = −0.39, *p* < 0.05). *Intestinimonas* and *Granulicatella* were positively correlated with azelaic acid, 3-methyladipic acid, 3-tert-butyladipic acid, and 16-hydroxypalmitic acid (0.38 < R < 0.50, *p* < 0.05), and negatively correlated with N-acetyl-1,4-diaminobutane (−0.54 < R < −0.49, *p* < 0.05). Additionally, an increase in *Granulicatella* was associated with downregulation of several arginine metabolic pathway intermediates, including L-arginine, N1-acetylspermine, N-acetyl-1,4-diaminobutane, and L-citrulline (−0.49 < R < −0.37, *p* < 0.05), potentially leading to metabolic disturbances in arginine and spermidine metabolism.

Fecal metabolites are absorbed into the bloodstream via the gut, resulting in variations in serum metabolite profiles and influencing host metabolic processes. Among the differentially abundant serum metabolites, 3,4-sulfocatechol was significantly positively correlated with *Bacteroides*, *Lactobacillus*, *Marvinbryantia*, and *Ruminococcaceae UCG-002* (0.38 < R < 0.41, *p* < 0.05). Tetradecanedioic acid exhibited a positive correlation with the *[Eubacterium] ventriosum group*, *Ruminococcaceae UCG-002*, and *Lactobacillus* (0.41 < R < 0.44, *p* < 0.05). *Granulicatella* showed significant positive correlations between 2-hydroxyhippuric acid and *Bacteroides* as well as *Marvinbryantia* (0.44 < R < 0.45, *p* < 0.05), while indole-3-acrylic acid had a significant positive correlation with *Granulicatella* (R = 0.37, *p* < 0.05). Additionally, behenic acid and arachidonic acid were significantly positively correlated with *Ruminococcus 1* (0.41 < R < 0.44, *p* < 0.05).

In summary, *Paraprevotella*, *Ruminococcus*, *Marvinbryantia*, and *Granulicatella*, along with the presence of metabolites such as azelaic acid, 3-tert-butyladipic acid, 3-methyladipic acid, and N-acetyl-1,4-diaminobutane in feces samples, may serve as potential biomarkers for the diagnosis of OP patients.

### Effects of different doses of *L. fermentum* CCFM1126 on bone structure of OP rats

**Figure 3.**
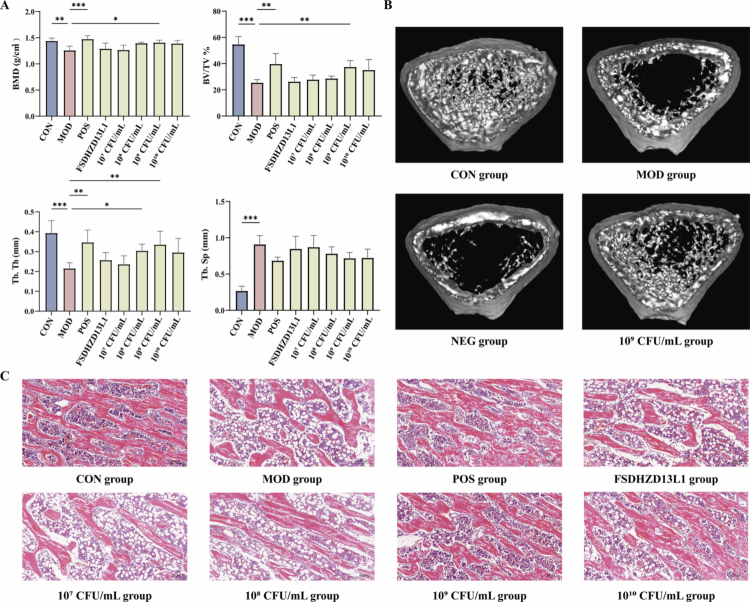
Effects of *L. fermentum* on bone structure of OP rats. (A) main parameters of femur structure, using non-parametric Kruskal–Wallis test, *, **, and *** indicate statistical significance, corresponding to *p* < 0.05, <0.01, and <0.001, respectively. (B) Bone microstructure. (C) Femoral pathology (50×).

To further investigate the optimal dose of *L. fermentum* CCFM1126 for improving bone structure, we systematically evaluated the effects of four different doses of CCFM1126 on OP rats and compared these outcomes with those of E_2_ and negative control strains *L. fermentum* FSDHZD13L1. As illustrated in Figure 3A and B (cropped region outlined in the corresponding raw image provided in Supplementary Figure S2), compared to the CON group, the MOD group exhibited decreased BMD, BV/TV, and Tb.Th, increased Tb.Sp, with a sparser bone microstructure. Following E_2_ intervention in rats within the MOD group, improvements were observed in femoral BMD and BV/TV, with a clearer mesh structure, increased Tb.Th, closer distribution, and more orderly arrangement. *L. fermentum* CCFM1126 at a concentration of 10^9^ CFU/mL demonstrated a superior regulatory effect on bone structure, with significant increases observed in BMD, BV/TV, and Tb.Th (*p* < 0.05). In contrast, FSDHZD13L1 at 10^9^ CFU/mL did not show any significant differences in bone structural indices compared to the MOD group (*p* > 0.05). Additionally, CCFM1126 at 10^8^ CFU/mL exhibited some improvement in Tb.Th.

After HE staining and microscopic examination, we conducted an in-depth analysis of the pathological structure of femurs in OP rats. As illustrated in Figure 3C (cropped region outlined in the corresponding raw image provided in Supplementary Figure S3), varying degrees of tissue damage and alterations were observed across different experimental groups. Consistent with the CT imaging findings, the MOD group exhibited a reduction in bone mass, disruption of the lamellar structure, and the presence of OP and trabecular separation. Additionally, we observed a certain degree of soft tissue injury and fibrotic response within the MOD group. This indicates the presence of a chronic inflammatory response surrounding the femur or other repair processes initiated by trauma. Following the administration of E_2_ or *L. fermentum* CCFM1126, the pathological condition of the bone was ameliorated. Specifically, low-dose CCFM1126 can mitigate the inflammatory response in the femur, whereas medium-to-high doses of CCFM1126 can significantly enhance the lamellar structure of the femur and reverse OP induced by estrogen deficiency in OP rats.

### Effects of different doses of *L. fermentum* CCFM1126 on gut microbiota in OP rats

**Figure 4.**
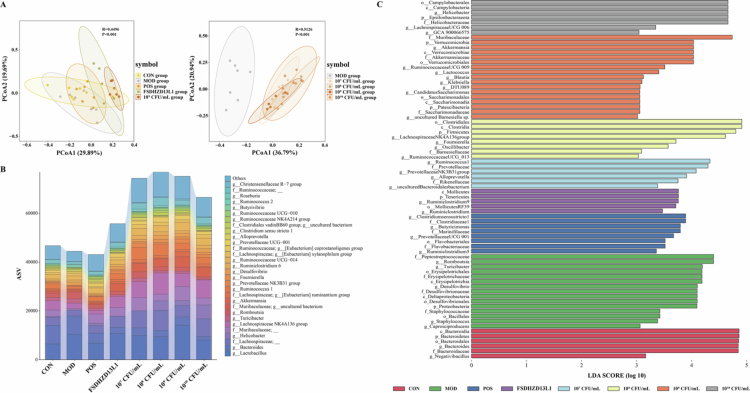
Effects of *L. fermentum* on gut microbiota of OP rats. (A) PCoA based on Bray–Curtis dissimilarity, illustrating overall differences in gut microbial community structure among experimental groups. Each point represents an individual sample, and ellipses indicate the 95% confidence interval for each group. The statistical significance of separation between groups was assessed by ANOSIM. (B) Stacked bar plot showing the relative abundance of dominant gut microbial genera across different experimental groups. Only genera with mean relative abundance above the threshold are displayed. (C) LEfSe identifying differentially abundant taxa among groups. Taxa with an LDA score > 3.0 and *p* < 0.05 were considered significantly discriminative.

The dose-relationship between *L. fermentum* regulation of gut microbiota was investigated in OP models. As illustrated in Figure S4, there was no significant alteration in the *α* diversity of gut microbiota in rats within the MOD group relative to the CON group. However, *L. fermentum* CCFM1126 significantly increased the α diversity of gut microbiota in OP rats at both low and high doses (*p* < 0.05). In Figure 4A, PCoA was employed to evaluate β diversity among the groups. The analysis revealed partial overlap between the CON group and other groups, indicating varying degrees of similarity in gut microbiota structure. To elucidate the dose-dependent effects of CCFM1126 on gut microbiota in OP rats, we compared the MOD group with four different dosages of CCFM1126. The results demonstrated distinct separation in principal component analysis between the intervention groups and the MOD group. Subsequently, we evaluated the impact of varying doses of *L. fermentum* CCFM1126 on the abundance and composition of gut microbiota in OP rats. We further investigated the potential of CCFM1126 to modulate gut microbiota by analyzing differences at the genus level. As illustrated in Figure 4B and C, different doses of CCFM1126 significantly altered the gut microbial profile of OP rats. The observed changes in overall diversity may be attributed to the increase or decrease in specific bacterial populations. At the genus level, compared with the CON group, the relative abundances of *Negativibacillus*, *Ruminococcaceae NK4A214 group*, and *Peptococcus* were significantly reduced. Conversely, *Corynebacterium 1*, *Rothia*, *Enterorhabdus*, and *Staphylococcus* showed significant increases. Following CCFM1126 intervention, *Bacteroides* decreased markedly, while *Lactobacillus*, *Akkermansia*, *Dorea*, *Blautia*, *Ruminococcaceae UCG-009*, *Ruminococcaceae UCG-013*, *Klebsiella*, *Lachnospiraceae NK4A136 group*, *Butyricicoccus*, *Butyricimonas*, and *Butyrivibrio* increased significantly.

### Effect of *L. fermentum* CCFM1126 on proliferation of MC3T3-E1 cells

**Figure 5.**
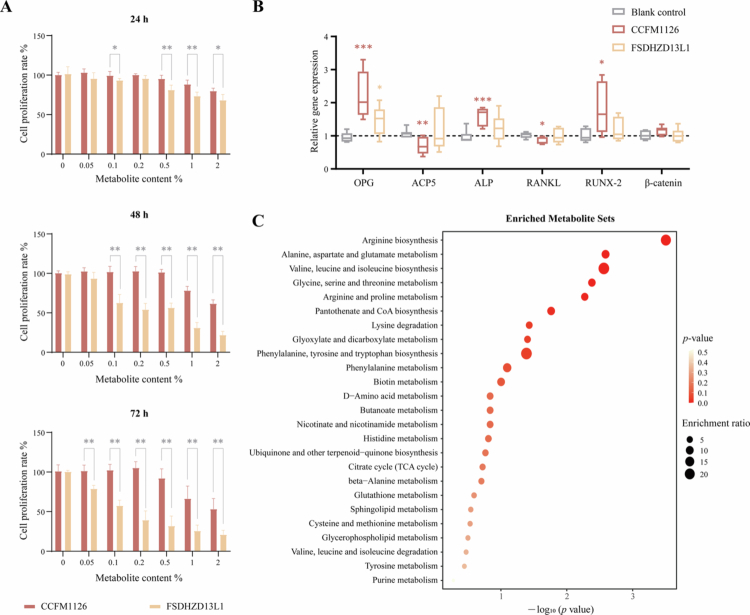
Comparison of metabolites between *L. fermentum* CCFM1126 and FSDHZD13L1 *in vitro*. (A) Effect of *L. fermentum* metabolites on proliferation of MC3T3-E1 cells. (B) Effect of *L. fermentum* metabolites on gene expression in MC3T3-E1 cells. (C) Differential metabolites of *L. fermentum*. *, **, and *** indicate statistical significance, corresponding to *p* < 0.05, <0.01, and <0.001, respectively.

We conducted a comparative analysis between *L. fermentum* CCFM1126, a strain possessing the capability to alleviate OP, and the negative control strain *L. fermentum* FSDHZD13L1, which does not exhibit this effect. The objective of this comparison was to further elucidate the potential material basis by which *L. fermentum* may mitigate OP. MC3T3-E1 cells were treated with varying concentrations (0%, 0.05%, 0.1%, 0.2%, 0.5%, 1%, and 2%) of CCFM1126 and FSDHZD13L1 supernatants, respectively. As showed in Figure 5A, cell proliferation decreased as the concentration of metabolic supernatant increased. Within 72 h, the addition of 0% to 0.5% CCFM1126 metabolic supernatant exhibited minimal toxicity to cells, indicating a safe dosage range. Conversely, at 48 and 72 h, FSDHZD13L1 metabolic supernatants at concentrations exceeding 0.1% significantly reduced cell proliferation, potentially attributable to variations in the metabolite profiles between the two strains. Additionally, at 72 , a 0.5% metabolic supernatant from both strains affected cell proliferation. Therefore, after comprehensive evaluation, a 0.1% concentration of the strain supernatant was selected for subsequent cell intervention experiments.

### Effect of *L. fermentum* CCFM1126 on gene expression in MC3T3-E1 cells

We examined the impact of the metabolic supernatants of *L. fermentum* CCFM1126 and FSDHZD13L1 on the expression of bone-related genes in MC3T3-E1 pre-osteoblast cells derived from mouse embryos. The relative mRNA expression levels of OPG, ACP5, ALP, RANKL, RUNX-2, and β-catenin in MC3T3-E1 cells treated with 0.1% *L. fermentum* metabolic supernatant for 24 h are presented in Figure 5B. Compared to the blank control, both CCFM1126 and FSDHZD13L1 significantly increased the mRNA expression of the OPG gene but had no significant effect on β-catenin gene expression. Additionally, the metabolic supernatant of CCFM1126 significantly upregulated the expression of ALP and RUNX-2 genes while downregulating the expression of the RANKL gene, effects not observed with FSDHZD13L1.

### Metabolites of *L. fermentum* CCFM1126 may be a potential key substance in alleviating OP

By comparing the metabolites of *L. fermentum* CCFM1126 with those of FSDHZD13L1, we explored the potential key substances responsible for alleviating OP in *L. fermentum*. As illustrated in Figure 5C and Table 4, after applying screening criteria (VIP > 1, FC > 1.2 or FC < 0.83, *p* < 0.05), CCFM1126 significantly up-regulated 26 differential metabolites and down-regulated 17 others. Compared to FSDHZD13L1, CCFM1126 exhibited higher metabolism of D-2-hydroxyglutaric acid, L-aspartate, 3-hydroxybutyric acid, ornithine, L-glutamic acid, and choline. Notably, *L. fermentum* CCFM1126 also increased the fecal choline content in subjects, which may be attributed to its superior ability to synthesize choline compared to FSDHZD13L1.

**Table 4.** Differential metabolites of *L. fermentum* CCFM1126 and FSDHZD13L1.

Differential metabolites	FC	*p-*value	VIP	Change
(+/−)9,10-dihydroxy-12Z-octadecenoic acid	3.246	0.0000	1.407	↑
D-α-hydroxyglutaric acid	2.180	0.0000	1.400	↑
L-aspartic acid	1.975	0.0000	1.213	↑
D-(−)-mannitol	1.973	0.0000	1.476	↑
2-hydroxyvaleric acid	1.944	0.0000	1.364	↑
N-acetylaspartic acid	1.827	0.0000	1.265	↑
Proline	1.658	0.0015	1.400	↑
Threonine	1.591	0.0000	1.058	↑
3-hydroxybutyric acid	1.584	0.0001	1.029	↑
Ornithine	1.321	0.0002	1.288	↑
DL-lysine	1.255	0.0005	1.076	↑
Citric acid	1.252	0.0019	1.362	↑
L-tyrosine	1.248	0.0015	1.162	↑
2-furoic acid	1.248	0.0016	1.283	↑
D-(+)-tryptophan	1.247	0.0029	1.027	↑
Valylproline	1.242	0.0010	1.022	↑
L-glutamic acid	1.239	0.0004	1.104	↑
4-hydroxybenzaldehyde	1.230	0.0006	1.124	↑
Choline	1.224	0.0006	1.373	↑
Acetophenone	1.220	0.0020	1.162	↑
DL-carnitine	1.218	0.0007	1.050	↑
3-[(4-hydroxyphenyl) methyl]-octahydropyrrolo[1,2-a] pyrazine-1,4-dione	1.213	0.0041	1.199	↑
Isoleucine	1.210	0.0002	1.147	↑
N-acetyl-L-phenylalanine	1.209	0.0016	1.173	↑
Valeric acid	1.208	0.0016	1.458	↑
3-(propan-2-yl)-octahydropyrrolo[1,2-a] pyrazine-1,4-dione	1.200	0.0082	1.043	↑
Bis(4-ethylbenzylidene) sorbitol	0.013	0.0000	1.474	↓
Glycyl-L-leucine	0.081	0.0000	1.397	↓
16-Hydroxyhexadecanoic acid	0.096	0.0000	1.477	↓
L-(+)-Arginine	0.249	0.0000	1.254	↓
2-hydroxy-4-methylthiobutanoic acid	0.317	0.0000	1.481	↓
PEG monooleate n8	0.330	0.0000	1.433	↓
Leucylproline	0.385	0.0000	1.471	↓
Oleic acid alkyne	0.401	0.0000	1.434	↓
PEG monooleate n7	0.446	0.0000	1.389	↓
PEG monooleate n6	0.593	0.0021	1.320	↓
L-serine	0.660	0.0000	1.452	↓
Guanine	0.727	0.0002	1.426	↓
Pantothenic acid	0.739	0.0000	1.456	↓
DL-4-hydroxyphenyllactic acid	0.759	0.0006	1.453	↓
L-threonic acid	0.784	0.0175	1.289	↓
Prolylleucine	0.800	0.0015	1.436	↓
Hexadecanamide	0.818	0.0100	1.396	↓

Note: "↑" indicates upward adjustment, "↓" indicates downward adjustment.

## Discussion

In recent years, the relationship between gut microbiota and bone metabolism has emerged as a focal point of research. Studies have demonstrated that gut microbiota is intricately linked with bone loss.^32^^,^^33^ The stability of the gut microbiome is essential for maintaining bone metabolic equilibrium, and the interaction between microbiota and the host modulates the host's capacity to maintain normal bone metabolic pathways.^34^ Disruptions in the balance of the gut microbiome can impair the gut immune response, compromise gut permeability, and promote the production of osteoclastogenic factors, leading to bone disorders.^35^^,^^36^ Moreover, OP may also interfere with the structure and metabolic activities of the gut microbiota as it progresses.^37^^,^^38^ Previous studies have revealed that alterations in Lactobacillus species are associated with bone loss.^26^ Specifically, different levels of Lactobacillus exhibit heterogeneity across various bone health populations: *Lactobacillus amylovorus*, *Lactobacillus crispatus*, *L. fermentum*, and *Lactiplantibacillus plantarum* are prevalent in healthy individuals, whereas *Lactobacillus acidophilus* tends to be more abundant in those with OP individuals.^26^

At present, the development and application of probiotics offer a novel research paradigm for the prevention and treatment of OP. Probiotics can effectively promote bone formation and maintain bone health through multiple mechanisms: regulating gene expression in epithelial and immune cells, modulating metabolites of gut microbiota, optimizing gut mucosal barrier function, reducing gut pH to enhance calcium absorption, and combating harmful gut microbiota^39-42^ demonstrated that *Lactobacillus rhamnosus* GG can mitigate estrogen deficiency-induced OP by regulating gut microbiota and barrier function, stimulating a balanced Th17/Treg response in both the gut and bone. *Lactobacillus helveticus* ATCC 27558 enhances bone health in ovariectomized rats by modulating bone remodeling, which is achieved through promoting bone formation while simultaneously reducing bone resorption.^21^ *Lactiplantibacillus plantarum* AR495 inhibits bone resorption and ameliorates ovariectomy-induced gut inflammatory response by modulating the receptor activator of nuclear factor κB/RANK ligand/osteoprotegerin (RANK/RANKL/OPG) pathway in ovariectomized mice.^43^ Simultaneously, *L. plantarum* AR495 improved the stability of the gut microbiota in these mice, resulting in a higher abundance of bacteria that produce short-chain fatty acids and increased levels of short-chain fatty acids in the feces.^43^ Supplementation with *Lactobacillus reuteri* NCIMB 30242 has been shown to significantly increase serum vitamin D levels and improve calcium absorption in healthy subjects.^44^ A randomized controlled trial showed that elderly women with BMD issues experienced significant benefits from continuous supplementation of *L. reuteri* ATCC PTA 6475 over a year.^25^ This approach effectively addressed gut microbiota imbalances and reversed the worsening of gut inflammatory conditions, ultimately exerting a positive impact on bone metabolism. Additionally, both *L. reuteri* ATCC PTA 6475 and *L. rhamnosus* GG effectively mitigated trabecular bone loss in OP mice induced by glucocorticoid-induced gut dysbiosis and barrier dysfunction.^45^ Consequently, incorporating probiotics into daily diets may serve as a promising strategy to prevent or ameliorate OP.

Gut microbiota metabolites encompass a diverse array of compounds generated through the metabolic activities of gut microbiota within the human gastrointestinal tract. These metabolites constitute approximately 10% of the total body metabolites and primarily consist of short-chain fatty acids, bile acids, choline metabolites, indole derivatives, polyamines, and phytoestrogens. They are recognized for their significant physiological roles and may serve as potential, specific targets for future interventions in bone metabolism.^14^ We conducted a comprehensive analysis of the serum and fecal metabolomics in OP patients treated with CCFM1126, and analyzed the association between key metabolites of CCFM1126 and differential metabolites of probiotic subjects, offering valuable insights into the molecular mechanisms and material basis underlying the efficacy of CCFM1126 in alleviating OP.

*L. fermentum* CCFM1126 was observed to increase the choline content in the feces of subjects, potentially due to its capacity for choline synthesis. Compared to FSDHZD13L1, CCFM1126 demonstrates a higher rate of choline metabolism. Choline is an essential nutrient required throughout human life and plays a critical role in the synthesis of neurotransmitters such as acetylcholine, methyl donors like betaine, and phospholipids.^46^ Although small amounts of choline can be synthesized via the phosphatidylethanolamine N-methyltransferase pathway, most individuals need to ensure adequate choline levels through dietary intake to prevent deficiency.^47^ Choline is metabolized into trimethylamine (TMA) by gut microbiota; upon consumption of choline-rich foods or other trimethylamine-containing compounds, both gram-positive and gram-negative bacteria in the gut produce TMA.^47^ Elevated levels of TMA and its oxidized form, TMAO, have been associated with increased activity of Firmicutes and Proteobacteria,^48^ with the CutC/CutD gene cluster encoding choline TMA lyase and its activator protein playing a crucial role.^49^ CutC/CutD clusters are prevalent in a diverse array of gut microbiota, notably within Clostridium cluster XIVa and Eubacterium species, as well as in certain actinomycetes and Proteobacteria.^48^ A diet deficient in choline may induce alterations in the gut microbiota composition and contribute to health issues. A controlled trial demonstrated that variations in dietary choline levels influenced gut microbiome composition, with *γ*-Proteobacteria and Erysipelotrichi being associated with changes in hepatic fat content during choline consumption.^50^ The Hordaland Health Study revealed a positive correlation between dietary choline intake and BMD,^51^ suggesting that choline might be a potential therapeutic agent for OP prevention. Furthermore, research indicates that a choline-deficient diet can interfere with and disrupt the gut barrier.^52^ Methionine-choline deficiency downregulates the Wnt/β-catenin pathway, leading to disruption of the gut vascular barrier and upregulation of serous vesicle-associated protein-1.^53^^,^^54^ Additionally, ZO-1 expression is reduced in patients on a methionine-choline deficient diet, resulting in increased bacterial translocation from the gut to peripheral organs such as the liver, contributing to steatosis and lipid accumulation.^55^ In addition, the aspartic acid and ornithine generated through CCFM1126 metabolism may be associated with host arginine biosynthesis, potentially enhancing the arginine content in host feces. Studies have demonstrated that arginine can improve arthritis and inhibit inflammatory bone loss by modulating osteoclast energy metabolism.^56^^,^^57^ The metabolites produced by this strain, including citric acid, aspartic acid, N-acetyl-L-aspartic acid, and glutamic acid, may contribute to the up-regulation of the host β-alanine metabolic pathway, which could be linked to malonic acid biosynthesis. Furthermore, aspartate and glutamate were found to be involved in host histidine metabolism, leading to a significant increase in fecal histidine levels following CCFM1126 intervention compared to the placebo group.

Despite extensive research on the functional evaluation of probiotics in alleviating OP, clinical application remains inconsistent. Although *L. fermentum* CCFM1126 has been shown to influence bone metabolism and exhibit significant efficacy in the adjuvant treatment of OP,^27^ the optimal dose of CCFM1126 for OP remains undetermined. Therefore, we conducted an ovariectomy-induced OP model study, which revealed that the bone structure of the distal femur in OP rats could be restored when the bacterial solution concentration reached 10^8^–10^9^ CFU/mL of CCFM1126. Moreover, CCFM1126 entered the colon via the digestive tract, leading to significant changes in gut microbiota at concentrations of 10^7^ CFU/mL, with better effects observed at concentrations exceeding 10^9^ CFU/mL. These findings reinforce our previous research and provide a foundation for future clinical trials. After considering bacterial activity, production costs, and dose-effect relationships, a dose of 5 × 10^9^ CFU was selected for twice-daily clinical intervention based on dose conversion between rats and humans. The findings were in accordance with our hypotheses. Compared to the placebo group, CCFM1126 not only significantly mitigated BMD loss in patients but also promoted bone formation and reduced bone resorption to a notable extent.

The classical Wnt/β-catenin and RANK/RANKL/OPG pathways are recognized as pivotal in bone metabolic signaling, particularly under the influence of the gut microbiota. The Wnt/β-catenin pathway primarily governs bone formation, whereas the RANK/RANKL/OPG pathway predominantly regulates bone resorption.^20^^,^^58^ In the Wnt/β-catenin signaling pathway, Wnt proteins bind to frizzled receptors on the surface of osteoblasts, forming a complex with low-density lipoprotein receptor-related protein 5/6.^59^^,^^60^ This complex subsequently interacts with cytoplasmic dishevelled proteins, resulting in the accumulation of β-catenin and its nuclear translocation. The nuclear β-catenin activates the transcription and expression of downstream osteogenic genes. For instance, ALP gene upregulation not only enhances metabolism and calcium deposition but also accelerates the differentiation of MC3T3-E1 cells into mature osteocytes. Runt-related transcription factor-2 (RUNX-2) is another key regulator of genes associated with osteoblast maturation.^61^^,^^62^ Concurrently, OPG secreted by osteoblasts can competitively bind to the RANK receptor, thereby inhibiting RANKL-induced osteoclast differentiation and preventing excessive bone resorption.^61^ As shown in the results of cell experiments, CCFM1126 has been shown to alleviate OP, with its metabolites playing a crucial role in this process.

These findings remain speculative and require further validation through additional research. While gut metabolites have garnered attention for evaluating the systemic tissue regulation by gut microbiota, translating animal studies into clinical applications presents challenges such as metabolite selection, safety and efficacy assessments, and determining appropriate administration timing and dosages.^63^ The swift progress in metabolomics and other innovative technologies could aid in investigating the metabolites and pathways associated with the regulation of bone metabolism and the maintenance of bone health.^3^^,^^64^^,^^65^ This can provide fresh perspectives and methods for the prevention and treatment of OP as well as for enhancing conditions related to bone metabolism.

Several limitations of this study should be acknowledged. We recognize that the sample size of this trial was relatively small; however, this reflects the exploratory, early-phase nature of the study, which is suitable for generating preliminary clinical and mechanistic insights. Despite this limitation, the consistency of the observed trends across clinical outcomes, animal models, *in vitro* experiments, and metabolomic analyses provides convergent evidence supporting the potential role of *L. fermentum* CCFM1126 in modulating bone metabolism. Future studies with larger, adequately powered cohorts are warranted to confirm these findings, refine dosing strategies, and further explore the underlying mechanisms.

## Conclusion

Our findings provide preliminary evidence that CCFM1126 may enhance bone formation and reduce bone resorption in individuals with osteoporosis, with choline emerging as a potential functional mediator and biomarker. These results underscore the feasibility and promise of targeted probiotic interventions for bone health. Future studies should aim to systematically elucidate the functional genes and metabolic pathways associated with choline metabolism in CCFM1126, as well as determine whether these mechanisms extend to other *L. fermentum* strains. Collectively, this work lays a solid foundation for larger, adequately powered, multi-center trials to confirm efficacy, optimize dosing strategies, and translate these insights into practical preventive and therapeutic applications for osteoporosis.

## Supplementary Material

Supplementary material The supplementary material.docx

## Data Availability

The data supporting the findings are available in the article and its supplementary materials. 16S rRNA gene sequencing data of gut microbiota have been deposited in the NCBI SRA under BioProject accession PRJNA1282905 (https://www.ncbi.nlm.nih.gov/bioproject/PRJNA1282905) and PRJNA1283383 (https://www.ncbi.nlm.nih.gov/bioproject/PRJNA1283383).
