# Supplementary material for: Limosilactobacillus fermentum CCFM1126 attenuates osteoporosis by modulating the gut microbiota composition and fecal metabolites: A randomized, double-blind, placebo-controlled clinical trial
Source: Gut Microbes Rep. 2026 May 16;3(1):2669712. doi: 10.1080/29933935.2026.2669712 (PMC13182971; doi:10.1080/29933935.2026.2669712)
Supplement: Supplementary material — The supplementary material.docx [file KGMR_A_2669712_SM8189.docx]

**The supplementary material**
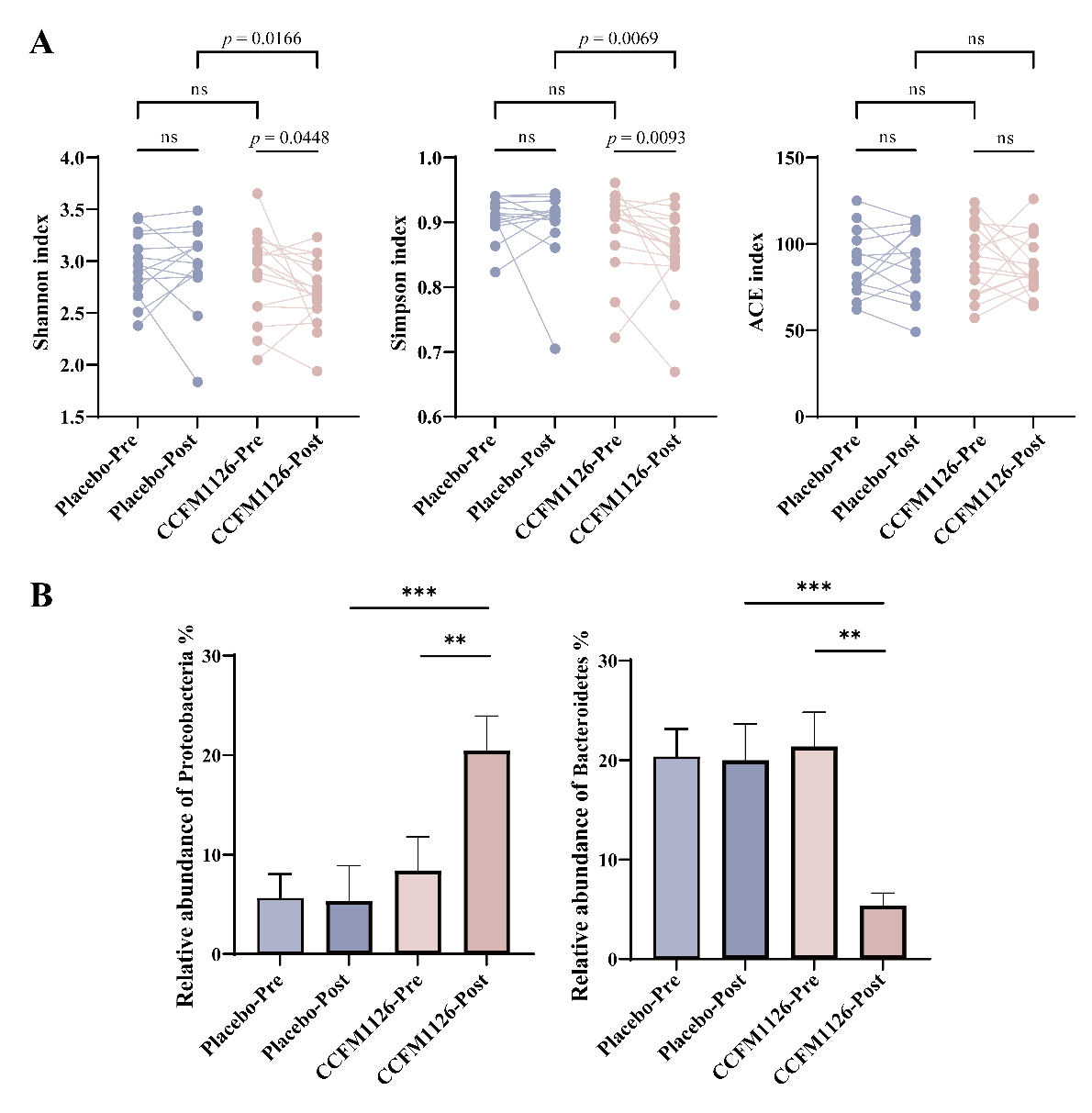


Figure S1 Effect of *L. fermentum* CCFM1126 on diversity and core microbial composition of gut microbiota in OP patients. (A) Diversity index of gut microbiota, each scatter represents a sample, and the line connects the baseline period and the end period of the same subject; "━" stands for Wilcoxon paired test, and "┌┐" stands for Wilcoxon unpaired test; ns indicates no significant difference, that is, *p* > 0.05. (B) Comparison of relative abundance of Proteobacteria and Bacteroidetes, Using Wilcoxon unpaired test, *, **, and *** indicates statistical significance, corresponding to *p* < 0.05, < 0.01, and < 0.001, respectively.


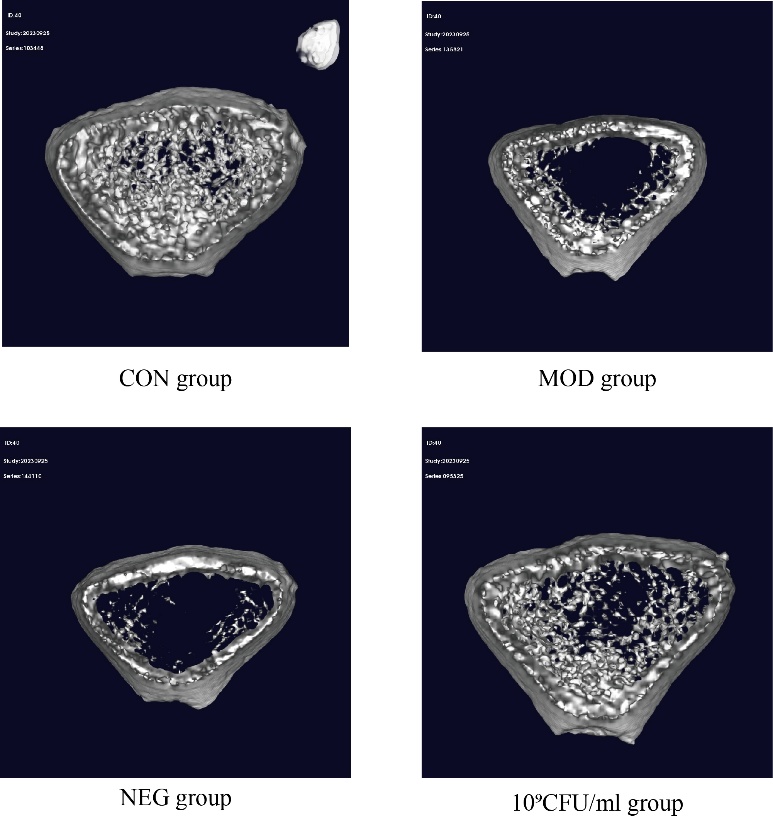


Figure S2 Unprocessed full-frame microscopy images for Figure 3B.


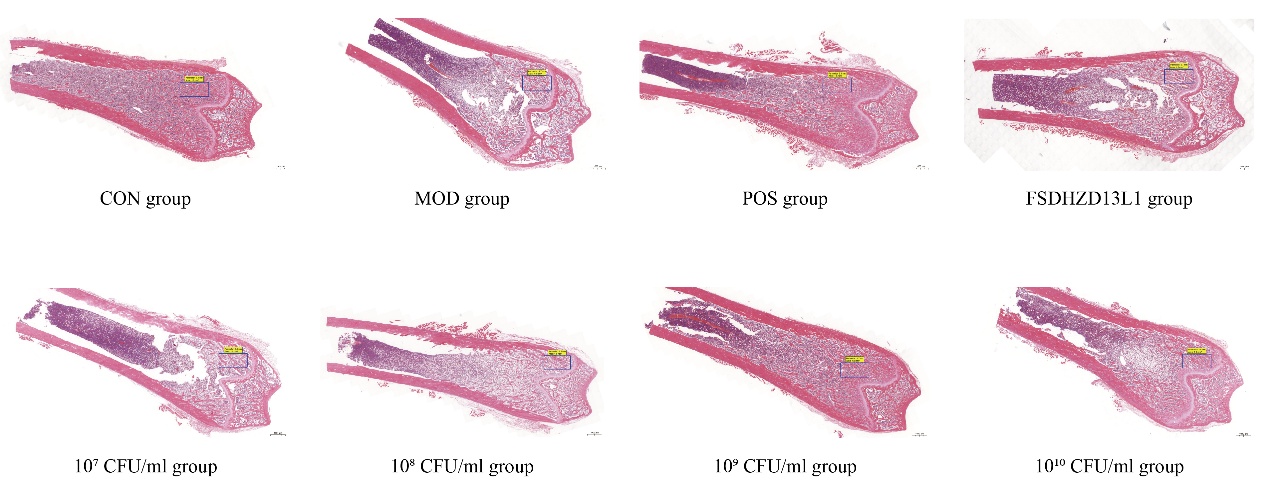


Figure S3 Unprocessed full-frame microscopy images for Figure 3C. Blue rectangles indicate the cropped regions used in the main article figures

**
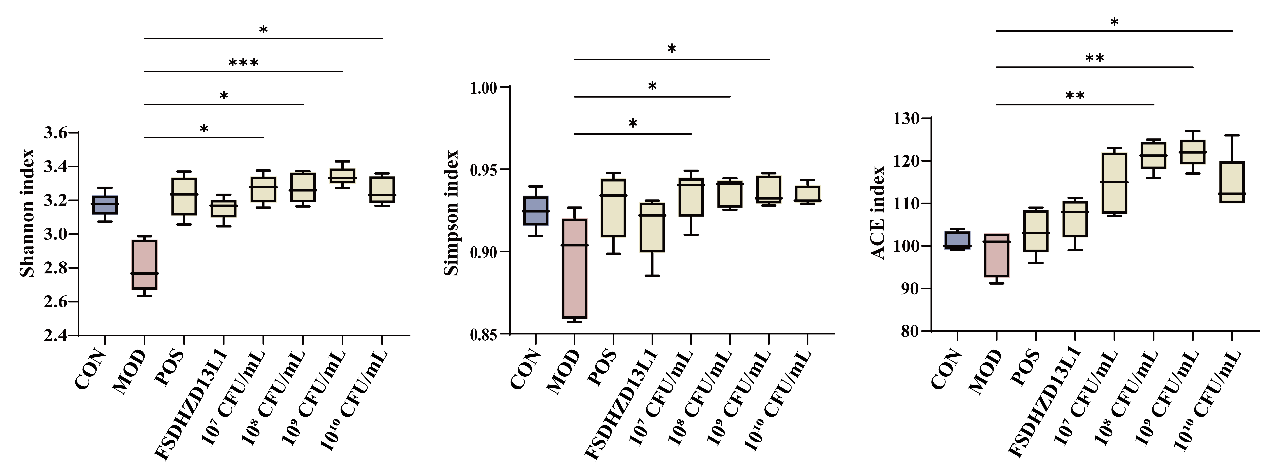
 Figure S4 Effects of *L. fermentum* CCFM1126 on diversity index of gut microbiota in OP rats.** Using non-parametric Kruskal-Wallis test, *, **, and *** indicates statistical significance, corresponding to *p* < 0.05, < 0.01, and < 0.001, respectively.

**Table S1 Subject demographics.**

| Numble | Gender | Age | BMI | Risk score | Group |
| --- | --- | --- | --- | --- | --- |
| 1 | Male | 68 | 23.44 | 2 | Placebo |
| 2 | Female | 75 | 25.32 | 6 | Placebo |
| 3 | Female | 79 | 22.89 | 6 | Placebo |
| 4 | Female | 56 | 22.72 | 4 | Placebo |
| 5 | Female | 53 | 27.34 | 1 | Placebo |
| 6 | Female | 57 | 31.11 | 5 | Placebo |
| 7 | Female | 68 | 21.26 | 3 | Placebo |
| 8 | Female | 68 | 24 | 2 | Placebo |
| 9 | Female | 59 | 21.63 | 1 | Placebo |
| 10 | Female | 54 | 19.63 | 5 | Placebo |
| 11 | Female | 65 | 18.67 | 4 | Placebo |
| 12 | Female | 70 | 24.24 | 4 | Placebo |
| 13 | Female | 81 | 25.39 | 4 | Placebo |
| 14 | Female | 50 | 21.5 | 6 | Placebo |
| 15 | Female | 60 | 21.48 | 4 | CCFM1126 |
| 16 | Female | 74 | 24.89 | 2 | CCFM1126 |
| 17 | Female | 70 | 21.23 | 6 | CCFM1126 |
| 18 | Female | 72 | 20.27 | 1 | CCFM1126 |
| 19 | Female | 76 | 27.64 | 2 | CCFM1126 |
| 20 | Female | 60 | 20.69 | 2 | CCFM1126 |
| 21 | Female | 58 | 23.44 | 5 | CCFM1126 |
| 22 | Female | 72 | 20.96 | 5 | CCFM1126 |
| 23 | Female | 60 | 23.44 | 2 | CCFM1126 |
| 24 | Female | 67 | 29.04 | 3 | CCFM1126 |
| 25 | Male | 74 | 27.63 | 2 | CCFM1126 |
| 26 | Female | 59 | 25.39 | 8 | CCFM1126 |
| 27 | Female | 59 | 23.73 | 2 | CCFM1126 |
| 28 | Female | 64 | 27.04 | 1 | CCFM1126 |
| 29 | Female | 70 | 28.91 | 4 | CCFM1126 |
| 30 | Female | 46 | 20.2 | 2 | CCFM1126 |
| 31 | Female | 60 | 21.5 | 6 | CCFM1126 |

**Table S2 Differences in feces metabolites between subjects in the placebo group and the probiotic group at the end of the intervention.**

| Differential metabolites | FC | *P* value | VIP | Change |
| --- | --- | --- | --- | --- |
| Losartan | 423.8 | 0.0412 | 1.012 | ↑ |
| Rosuvastatin | 158.7 | 0.0278 | 1.034 | ↑ |
| N1-Acetylspermine | 10.43 | 0 | 1.664 | ↑ |
| Malonic acid | 9.544 | 0.012 | 1.279 | ↑ |
| 2-Chlorobenzoic acid | 6.161 | 0.0246 | 1.097 | ↑ |
| 5-trans prostaglandin F2β | 5.217 | 0.0116 | 1.268 | ↑ |
| N-Acetylputrescine | 3.968 | 0.0011 | 1.536 | ↑ |
| L-Histidine | 3.878 | 0 | 2.101 | ↑ |
| Testosterone sulfate | 3.701 | 0.0002 | 1.445 | ↑ |
| (15Z)-9,12,13-Trihydroxy-15-octadecenoic acid | 3.519 | 0.0026 | 1.26 | ↑ |
| D- (+)-Maltose | 2.952 | 0.0001 | 2.202 | ↑ |
| Docosapentaenoic acid | 2.925 | 0.0024 | 1.37 | ↑ |
| 2,3-Dihydroxybenzoic acid | 2.921 | 0.0113 | 1.199 | ↑ |
| Choline | 2.853 | 0.0003 | 1.435 | ↑ |
| Uridine | 2.846 | 0.0159 | 1.594 | ↑ |
| DL-Arginine | 2.683 | 0.0185 | 1.515 | ↑ |
| 5-[(1S,2R,4aR)-5-(Hydroxymethyl)-1,2,4a-trimethyl-1,2,3,4,4a,7,8,8a-octahydro-1-naphthalenyl]-3-methylpentanoic acid | 2.568 | 0.027 | 1.091 | ↑ |
| Salicylic acid | 2.52 | 0.0096 | 1.186 | ↑ |
| Guanine | 2.456 | 0.0026 | 1.517 | ↑ |
| L- (+)-Citrulline | 2.443 | 0.0112 | 1.491 | ↑ |
| Tetradecanedioic acid | 2.279 | 0.0391 | 1.113 | ↑ |
| Gentisic acid | 2.133 | 0.0076 | 1.472 | ↑ |
| Glycyl-L-leucine | 2.074 | 0.0373 | 1.274 | ↑ |
| Tauroursodeoxycholic acid | 0.093 | 0.0004 | 2.017 | ↓ |
| Pentadecanoic acid | 0.112 | 0.0016 | 1.884 | ↓ |
| Palmitic acid | 0.138 | 0.0005 | 1.376 | ↓ |
| 3-Methyladipic acid | 0.156 | 0.002 | 1.777 | ↓ |
| Erucic acid | 0.169 | 0.0001 | 1.218 | ↓ |
| Taurochenodeoxycholic acid | 0.169 | 0.0008 | 1.794 | ↓ |
| 3,4-Dihydroxyphenylpropionic acid | 0.173 | 0.0285 | 1.208 | ↓ |
| Nervonic acid | 0.177 | 0 | 1.816 | ↓ |
| 16-Hydroxyhexadecanoic acid | 0.213 | 0.0001 | 1.799 | ↓ |
| 3-tert-Butyladipic acid | 0.247 | 0.0061 | 2.018 | ↓ |
| Stearic acid | 0.247 | 0.0002 | 2.101 | ↓ |
| Methylsuccinic acid | 0.266 | 0.006 | 2.381 | ↓ |
| (4-Phenoxyphenyl) 4-hydroxypiperidine-1-carboxylate | 0.276 | 0.023 | 1.113 | ↓ |
| Azelaic acid | 0.298 | 0.0048 | 1.859 | ↓ |
| 3-Acetyl-11-keto-β-boswellic acid | 0.339 | 0.0072 | 2.073 | ↓ |
| Suberic acid | 0.351 | 0.0367 | 1.763 | ↓ |
| Deoxycholic acid | 0.379 | 0.0331 | 1.133 | ↓ |
| Arachidic acid | 0.381 | 0 | 2.122 | ↓ |
| Arjungenin | 0.423 | 0.0005 | 2.078 | ↓ |
| Cholic acid | 0.447 | 0.0182 | 1.759 | ↓ |
| 3-[4-methyl-1-(2-methylpropanoyl)-3-oxocyclohexyl] butanoic acid | 0.447 | 0.0393 | 1.761 | ↓ |
| Irbesartan | 0.448 | 0.0322 | 1.398 | ↓ |
| D-α-Hydroxyglutaric acid | 0.465 | 0.0143 | 1.702 | ↓ |
| 2,3,4,9-Tetrahydro-1H-β-carboline-3-carboxylic acid | 0.484 | 0.0159 | 1.773 | ↓ |
| Docosahexaenoic acid ethyl ester | 0.499 | 0.0268 | 1.409 | ↓ |

**Table S3 Feces metabolites of probiotic group subjects before and after intervention.**

| Differential metabolites | FC | *P* value | VIP | Change |
| --- | --- | --- | --- | --- |
| Genistein | 27.4 | 0.0053 | 1.199 | ↑ |
| Acesulfame | 22.33 | 0.0479 | 1.293 | ↑ |
| Malonic acid | 11.37 | 0.0169 | 1.902 | ↑ |
| Daidzein | 5.534 | 0.0074 | 1.972 | ↑ |
| Guanine | 4.055 | 0.0018 | 2.357 | ↑ |
| Formononetin | 3.849 | 0.0009 | 2.144 | ↑ |
| Uridine | 2.761 | 0.0116 | 1.853 | ↑ |
| Acetylcholine | 2.616 | 0.0153 | 1.255 | ↑ |
| N1-Acetylspermine | 2.154 | 0.0112 | 1.896 | ↑ |
| Choline | 2.069 | 0.0305 | 2.243 | ↑ |
| L- (+)-Citrulline | 2.014 | 0.017 | 1.839 | ↑ |
| Metformin | 0.001 | 0.0144 | 1.285 | ↓ |
| Clopidogrel | 0.008 | 0.049 | 1.525 | ↓ |
| Stercobilin | 0.257 | 0.0459 | 1.318 | ↓ |
| Nervonic acid | 0.262 | 0.0035 | 2.294 | ↓ |
| Tauroursodeoxycholic acid | 0.334 | 0.0059 | 1.734 | ↓ |
| Cholecalciferol | 0.361 | 0.0088 | 1.945 | ↓ |
| Erucic acid | 0.387 | 0.0078 | 1.28 | ↓ |
| L-Tyrosine | 0.406 | 0.0407 | 1.726 | ↓ |
| 16-Hydroxyhexadecanoic acid | 0.412 | 0.0136 | 2.098 | ↓ |
| Palmitic acid | 0.421 | 0.0431 | 1.768 | ↓ |
| Arachidic acid | 0.457 | 0.0278 | 1.859 | ↓ |

Table S-4 Differences in serum metabolites between subjects in the placebo group and the probiotic group at the end of the intervention.

| Differential metabolites | FC | *P* value | VIP | Change |
| --- | --- | --- | --- | --- |
| Irbesartan | 77.01 | 0.0716 | 1.078 | ↑ |
| Estazolam | 11.57 | 0.067 | 1.134 | ↑ |
| Cholic acid | 3.384 | 0.1092 | 1.463 | ↑ |
| (±)5-iPF2α-VI | 3.122 | 0.032 | 1.468 | ↑ |
| β-Muricholic acid | 3.113 | 0.1199 | 1.393 | ↑ |
| PEG n11 | 2.119 | 0.0496 | 1.115 | ↑ |
| N-{4-[(2R,3R)-3-(Hydroxymethyl)-4-isopropyl-5-oxo-2-morpholinyl] phenyl}-2-methoxyacetamide | 2.072 | 0.0666 | 1.109 | ↑ |
| (+/-)9(10)-EpOME | 2.058 | 0.0226 | 1.658 | ↑ |
| (±)7(8)-DiHDPA | 1.825 | 0.0085 | 1.354 | ↑ |
| Bis(4-ethylbenzylidene) sorbitol | 2.934 | 0.0481 | 1.598 | ↑ |
| Gliclazide | 0.01 | 0.6387 | 1.003 | ↓ |
| Clopidogrel carboxylic acid | 0.015 | 0.1727 | 1.303 | ↓ |
| Pioglitazone | 0.021 | 0.5944 | 1 | ↓ |
| Triethyleneglycol bis(2-ethylhexanoate) | 0.047 | 0.6239 | 1.022 | ↓ |
| Phenol | 0.064 | 0.329 | 1.458 | ↓ |
| N1-(4-isopropylphenyl)-4-(2,5-dioxotetrahydro-1H-pyrrol-1-yl) benzene-1-sulfonamide | 0.073 | 0.6554 | 1.004 | ↓ |
| Salicylic acid | 0.085 | 0.3891 | 1.453 | ↓ |
| 3-(2-morpholinophenyl)-2-(phenylsulfonyl)acrylonitrile | 0.098 | 0.6961 | 1.004 | ↓ |
| 18-β-Glycyrrhetinic acid | 0.105 | 0.6185 | 1.024 | ↓ |
| 2-Hydroxyhippuric acid | 0.028 | 0.112 | 1.448 | ↓ |
| PB-22 N-(4-Hydroxypentyl)-3-carboxyindole metabolite | 0.372 | 0.4359 | 1.047 | ↓ |
| 3,4-Dihydroxybenzenesulfonic acid | 0.382 | 0.0953 | 1.75 | ↓ |
| Docosanoic acid | 0.468 | 0.0516 | 1.283 | ↓ |
| Hippuric acid | 0.484 | 0.8381 | 1.068 | ↓ |
| N-({(1S,4S,6S)-6-Isopropyl-3-methyl-4-[2-oxo-2-(1-pyrrolidinyl) ethyl]-2-cyclohexen-1-yl} methyl)-2-pyrazinecarboxamide | 0.553 | 0.0588 | 1.791 | ↓ |
| (2S,5aS,8aR)-6-Benzyl-1-methyl-2-[3-(4-morpholinyl)-3-oxopropyl] octahydropyrrolo[3,2-E] [1,4] diazepin-5(2H)-one | 0.553 | 0.1174 | 1.956 | ↓ |
| Arachidic acid | 0.561 | 0.0718 | 1.595 | ↓ |
| Risperidone | 0.597 | 0.1993 | 1.368 | ↓ |
| Tetradecanedioic acid | 0.6 | 0.5773 | 1.241 | ↓ |
| 4-{5-[(4-methylphenyl) methylene]-4-oxo-2-thioxo-1,3-thiazolan-3-yl}-N-(1,3-thiazol-2-yl) benzenesulfonamide | 0.611 | 0.1232 | 1.434 | ↓ |
| 1-(4-Methyl-1-piperazinyl)-2-[(3R,4S)-3-{[5-(phenoxymethyl)-1,2-oxazol-3-yl] methyl}-4-piperidinyl] ethanone | 0.612 | 0.0285 | 1.763 | ↓ |
| Indole-3-acrylic acid | 0.625 | 0.2975 | 1.658 | ↓ |
| 3-{[4-(1,3-Benzodioxol-5-ylmethyl) piperazino] carbonyl}-6,7-dimethoxy-2H-chromen-2-one | 0.656 | 0.1134 | 1.962 | ↓ |
| Nervonic acid | 0.661 | 0.1221 | 1.593 | ↓ |

Table S-5 Serum metabolites of probiotic group subjects before and after intervention.

| Differential metabolites | FC | *P* value | VIP | Change |
| --- | --- | --- | --- | --- |
| Bis(4-ethylbenzylidene) sorbitol | 5.461 | 0 | 2.612 | ↑ |
| Deoxycholic acid | 2.975 | 0.0037 | 1.83 | ↑ |
| Stachydrine | 2.137 | 0.0139 | 2.13 | ↑ |
| Chenodeoxycholic Acid | 2.144 | 0.0324 | 1.437 | ↑ |
| 3-(2,6-Dioxocyclohexyl) propanenitrile | 1.932 | 0.1435 | 1.46 | ↑ |
| Docosahexaenoic acid ethyl ester | 1.904 | 0.0478 | 1.399 | ↑ |
| 18-β-Glycyrrhetinic acid | 1.791 | 0.0698 | 1.5 | ↑ |
| PEG n11 | 1.542 | 0.3179 | 1.003 | ↑ |
| 5-Hydroxydiclofenac | 0.014 | 0.0405 | 1.451 | ↓ |
| Aceclofenac | 0.14 | 0.1028 | 1.404 | ↓ |
| Theobromine | 0.219 | 0.2028 | 1.309 | ↓ |
| (3β,9ξ)-3,14-Dihydroxycarda-5,20(22)-dienolide | 0.417 | 0.0521 | 1.72 | ↓ |
| 2-Mercaptobenzothiazole | 0.452 | 0.2708 | 1.48 | ↓ |
| Linoleic Acid | 0.476 | 0.0489 | 1.508 | ↓ |
| 11,12-Epoxy-(5Z,8Z,11Z)-icosatrienoic acid | 0.523 | 0.3418 | 1.005 | ↓ |
| CP 47,497-C6-Homolog | 0.529 | 0.0955 | 1.544 | ↓ |
| 16-Hydroxyhexadecanoic acid | 0.531 | 0.016 | 1.163 | ↓ |
| Sphingosine (d18:1) | 0.536 | 0.0279 | 1.57 | ↓ |
| 3,4-Dihydroxybenzenesulfonic acid | 0.579 | 0.1843 | 1.049 | ↓ |
| Citric acid | 0.606 | 0.0238 | 1.408 | ↓ |
| Palmitoleic acid | 0.614 | 0.3212 | 1.025 | ↓ |
| (±)7(8)-DiHDPA | 0.666 | 0.0748 | 1.397 | ↓ |
